# Plasma anti-myosin autoantibodies in the diagnosis of necrotizing enterocolitis

**DOI:** 10.1007/s00431-023-05188-6

**Published:** 2023-09-16

**Authors:** Yuqiong Chen, Chaoting Lan, Weiyong Zhong, Kai Song, Zuyi Ma, Lihua Huang, Yun Zhu, Huimin Xia

**Affiliations:** 1 Department of Pediatrics, The First Affiliated Hospital of Jinan University, No. 613 West Huangpu Avenue, Tianhe District, Guangzhou, Guangdong CN 510630 China; 2 Provincial Key Laboratory of Research in Structure Birth Defect Diseaseand, Department of Pediatric Surgery, Guangzhou Women and Children’s Medical Center, Guangzhou Medical University, No.9 Jinsui Road, Zhujiang New Town, Tianhe District, Guangzhou, Guangdong CN 510623 China; 3 Department of Pediatrics, The First People’s Hospital of Chenzhou, Chenzhou, Hunan China

**Keywords:** Necrotizing enterocolitis, Diagnosis, Autoantibody, Myosin

## Abstract

We aimed to assess whether autoantibodies can be used as biomarkers for necrotizing enterocolitis (NEC) and applied for its early diagnosis. A prospective observational study was conducted in neonates with suspected NEC abdominal distension (the developmental study), which consisted of 50 neonates finally divided into NEC (*n* = 24) and non-NEC (*n* = 26) cohorts based on follow-up results. Serum samples were collected within 48 h of illness onset and used for screening NEC-associated plasma autoantibodies by autoantigen microarray. Additionally, we validated anti-myosin autoantibodies by enzyme-linked immunosorbent assay (ELISA) in an independent validation study, for which we selected plasma samples within 48 h of onset of NEC (*n* = 38) and samples of gestational age- and weight-matched controls (*n* = 13). Autoantigen microarray revealed that both IgG and IgM anti-myosin autoantibodies in plasma from neonates with NEC were significantly higher than those in neonates with other diagnoses. ELISA showed that plasma anti-myosin autoantibodies increased in the NEC cohort, with 1.5-fold higher levels than in the non-NEC cohort. Anti-myosin autoantibodies were able to distinguish NEC from non-NEC, achieving an area under the curve (AUC) of 0.8856 (95% confidence interval (CI): 0.7918–0.9795), with sensitivity of 81.58% and specificity of 76.93%. Plasma anti-myosin autoantibodies were significantly higher in all three subtypes of NEC (*P* < 0.0001 for NEC I; *P* = 0.0018 for NEC II; *P* = 0.0011 for NEC III), especially in NEC stage I than that in the non-NEC controls.

*Conclusion*: Anti-myosin autoantibodies may be applied as a promising diagnostic marker for NEC, especially for NEC stage I.

**Table:** 

**What is Known:** *• Intestinal damage and self-antigen exposure may lead to increased autoantibodies, and they are widely used as biomarkers for diagnosing inflammatory bowel disease.* *• Necrotizing enterocolitis (NEC) is a devastating disease with overwhelming inflammation and immune dysregulation.*	
**What is New:** *• Increased autoantibodies were present in patients with NEC, even before typical X-ray manifestations.* *• Anti-myosin autoantibodies may be applied as a promising diagnostic marker for NEC.*

**Supplementary Information:**

The online version contains supplementary material available at 10.1007/s00431-023-05188-6.

## Introduction

Necrotizing enterocolitis (NEC) is a devastating disease with high morbidity and mortality (15% to 30%) among preterm infants [1]. Early symptoms of NEC, such as abdominal distension and haematochezia, are nonspecific but progress rapidly, with intestinal necrosis and perforation [2]. Early diagnosis is challenging due to the overlap of signs and symptoms with other gastrointestinal disorders [1, 3]. The presence of portal venous gas in abdominal X-ray is a typical imaging feature of NEC, but it occurs in bell stage II or more [4]. Because of the limited diagnostic accuracy of laboratory tests and delayed imaging modalities, biomarkers that can sensitively identify infants at risk of developing NEC are needed.

The pathologic characteristics of NEC include defects in the intestinal epithelial barrier and overwhelming inflammation [5, 6]. Intestinal immaturity, mucosal damage, pathogenic microbial invasion, and inappropriate feeding can all lead to intestinal damage and an inflammatory cascade in newborns [7]. Hypoxia and infection in utero or during birth also increase the risk of NEC [8]. Previous studies have shown that damage to tissue cells can cause the production of autoantibodies [9]. Autoimmunity has been verified in inflammatory bowel disease and immune-mediated inflammatory diseases and has been linked to disease severity and activity [10]. However, the role of autoimmunity in the development of NEC remains unknown.

Myosin, a hexameric ATPase cellular motor protein with two heavy chains (MHCs) and four light chains (MLCs), is a sarcolemmal protein involved in the process of muscular contraction [11]. Myosin exists inside cells, and tissue damage and exposure to self-antigens may be caused by toxins, ischaemia, or inflammation [12]. Anti-myosin autoantibodies are widely used as diagnostic biomarkers, especially in heart diseases such as myocarditis [13], dilated cardiomyopathy [14], the Kawasaki disease [15], rheumatic fever [16], and myocardial ischaemia [17]. Intestinal injury, inflammation, and necrosis are important features of NEC. Therefore, we hypothesize that autoantibodies produced by antigen exposure are involved in the development of NEC.

In this study, we screened for activated autoantibodies using an autoantigen microarray in neonates with suspected NEC abdominal distension (the developmental study). We found that both IgG and IgM among anti-myosin autoantibodies in plasma from neonates with NEC were significantly higher than in neonates with other diagnoses. We speculated that elevated anti-myosin autoantibodies may predict the risk of developing NEC at an early stage. To assess whether anti-myosin autoantibodies can diagnose NEC in preterm infants, we measured concentrations of serum anti-myosin autoantibodies in an independent validation study. The results showed that anti-myosin autoantibodies are able to distinguish NEC from non-NEC, achieving an area under the curve (AUC) of 0.8856, with an AUC of 0.9457 for NEC stage I. Our data suggest that anti-myosin autoantibodies may serve as a biomarker for the diagnosis of NEC, especially in NEC stage I.

## Materials and methods

### Subjects

Both the developmental study (NEC = 24; non-NEC = 26) and validation study (NEC = 38; non-NEC = 13) were enrolled in 2019 and 2020, and NEC cohorts with onset time less than 48 h were enrolled. Both studies were prospective. According to bell staging criteria [1], the diagnosis of NEC was confirmed by more than three neonatologists and neonatal surgeons, who were blinded to autoantibodies testing. The study was approved by the Medical Ethics Committee of Guangzhou Women and Children’s Medical Center (No. 2018052406), and written consent was obtained from the participant's parents or legal guardian.

The developmental study included consecutively admitted neonates with sudden abdominal distension and a possible clinical result of NEC, and the serum samples were used to test for IgM and IgG autoantibodies. Eligible newborns were identified by neonatologists who were unaware of the study aims, with the following criteria fulfilled: (1) premature infants with a corrected gestational age of less than 44 weeks; (2) sudden abdominal distension, with haematochezia or positive fecal occult blood, and onset time less than 48 h; and (3) parental consent obtained. Newborns with the following conditions were excluded: (1) Apgar score < 5 at 5 min; (2) congenital malformations or inborn errors of metabolism; (3) maternal history of autoimmune diseases; and (4) incomplete clinical data. Finally, 50 neonates were included, and 24 were eventually diagnosed with NEC.

Additionally, the validation study was independent; samples were obtained from the hospital’s biobank, from which we selected plasma samples of diagnosed NEC preterm patients. And we only included plasma samples within 48 h of the onset of NEC (*n* = 38). NEC patients were stratified according to gestational age at birth. Next step, birth weight, and age at the time of blood test, and sex-matched preterm controls were selected, finally including 13 matched controls. The exclusion criteria were the same as those for the developmental study.

### Data collection and clinical assessment

Patient demographic and clinical information, including gestational age, birth weight, sex, delivery mode, fetal distress, feeding, diagnosis, and treatment, were obtained from electronic medical records. Clinical information was collected and checked by 2 independent clinicians. To avoid information bias, the clinicians were blinded to the subsequent autoantibody tests.

Additionally, blood specimens for autoantigen microarray were collected in EDTA vacuum tubes, and plasma was obtained by centrifugation at 1500 × rpm for 20 min at 4 °C within 2 h and again at 3000 × rpm for 15 min. The collected plasma was stored at − 80 °C. Blood samples for ELISA were peripheral blood samples taken for routine blood tests when the development of NEC was first suspected.

### Autoantigen microarray

We used an autoantigen microarray composed of 48 autoantigens and 8 calibration proteins to screen for human autoimmune antigen microarrays. The autoantigen microarrays were manufactured, hybridized, and scanned as previously described [18]. IgG and IgM autoantibody tests were performed at Yijin Biotechnology Co., Ltd.

### ELISA

Quantitative sandwich enzyme immunoassays for human anti-myosin heavy chain autoantibodies (MYHA) were performed as recommended by the manufacturer (Shanghai Zhenke Biotechnology Co., Ltd., ZK-15613).

### Statistical analysis

Statistical analysis of the data was conducted by employing R and GraphPad Prism 8.0 (GraphPad Software Inc., CA, USA). A normal distribution of the variables was tested using the Shapiro–Wilk test. Normalized data are described by the mean and standard deviation. Quantitative variables were analyzed utilizing the* t* test, and grade data were assessed using chi-square tests. Analysis of covariance models was used when comparing continuous variables adjusting for potential confounders. The significance level was set at *P* < 0.05, and all* P* values were obtained from two-sided tests.

For autoantigen microarray analysis, normalization was executed by implementing the robust-linear-mode (RLM) method. M-statistics were applied for differential markers detection. The differential markers were subjected to cluster analysis by R package of “pheatmap.”

A receiver operating characteristic (ROC) curve provided the AUC for diagnostic value analysis. Diagnostic accuracy was evaluated through sensitivity and specificity measurements.

## Results

### Characteristics of the subjects

**Fig. 1:**
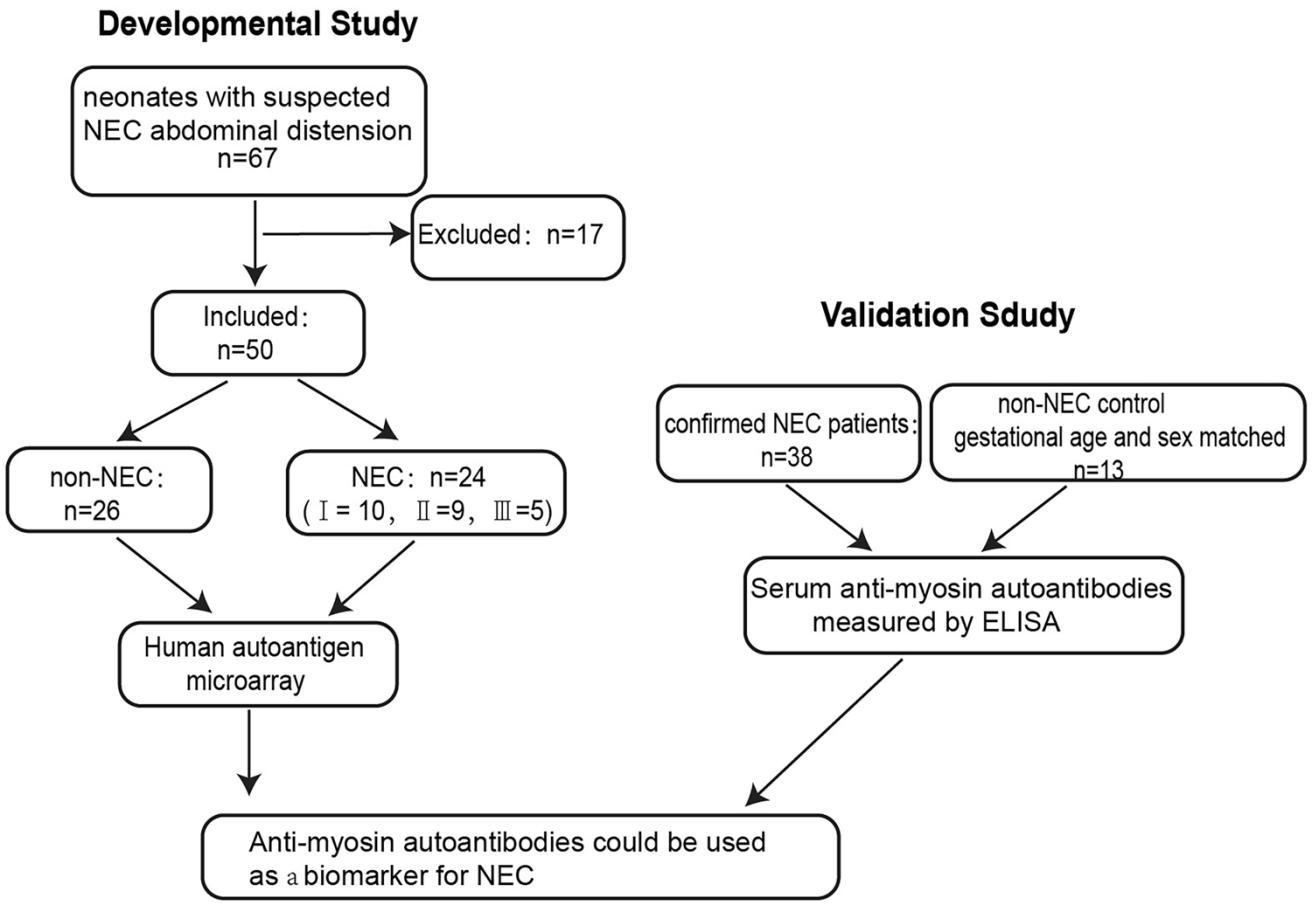
Flow chart of subject enrolment

In the developmental study, a total of 67 neonates were enrolled, and 17 were excluded due to low Apgar scores or related medical history. Finally, 50 neonates were included, and 24 were eventually diagnosed with NEC. They were further classified into bell stages I (*n* = 10), II (*n* = 9), and III (*n* = 5). The remaining 26 patients were assigned to the non-NEC cohort, and they were diagnosed with feeding intolerance (*n* = 13, including milk protein allergies and lactose intolerance), Hirschsprung’s disease (*n* = 5), intestinal malrotation (*n* = 5), or gastrointestinal problems of unknown origin (*n* = 3). The flow chart of subject enrolment is shown in Fig. 1. There was no significant difference in birth weight, or sex, between the NEC and non-NEC cohorts (P > 0.05, Table 1), but gestational age was younger in the NEC cohort (30.02 ± 1.96 vs. 31.51 ± 2.02, *P* = 0.015).

**Table 1:** Patient characteristics of clinical cases

	Developmental study			Validation study		
	non-NEC	NEC	*P*	non-NEC	NEC	*P*
Gestational age (weeks)	31.51 ± 2.02	30.02 ± 1.96	0.015	30.21 ± 1.93	29.61 ± 1.525	0.323
Birth weight (g)	1403.85 ± 288.85	1279.17 ± 280.51	0.129	1159.23 ± 174.62	1177.63 ± 223.32	0.763
Sex (male, *N* (%))	18 (69.2)	14 (58.3)	0.423	7 (53.8)	25 (65.8)	0.442
Cesarean section (*N* (%))	16 (61.5)	15 (62.5)	0.944	8 (61.5)	21 (55.3)	0.693
Apgar score at 5 min	9.23 ± 0.951	9 ± 0.78	0.355	9.54 ± 0.52	9.45 ± 0.83	0.712
Antenatal steroids^a^ (*N* (%))	13 (50.0)	16 (66.7)	0.233	9 (69.2)	30 (78.9)	0.476
PROM^b^ > 18 h (N (%))	7 (26.9)	7 (29.2)	0.86	3 (23.1)	9 (23.7)	0.964
Intrapartum antibiotic prophylaxis for PROM (N (%))	5 (19.2)	4 (16.7)	0.814	2 (5.4)	7 (18.4)	0.804
Breastfeeding (N (%))	20 (76.9)	19 (79.2)	0.848	10 (76.9)	30 (78.9)	0.878
Early-onset sepsis (N (%))	1 (3.8)	2 (8.3)	0.504	0 (0)	1 (2.6)	0.555
Late-onset sepsis (N (%))	0 (0)	1	0.293	1 (7.7)	2 (5.3)	0.748
Age (day)	22.15 ± 12.18	28.67 ± 20.79	0.189	23.85 ± 13.54	24.03 ± 13.52	0.967
Total	26	24		13	38	

^a^Intramuscular steroid cycle in two doses of 12 mg over a 24-h period

^b^PROM, prelabor rupture of membranes

^c^Breast milk feeding means breast milk > 50%

*NEC* necrotizing enterocolitis

Data were expressed as $\overline{\mathrm x}$ ± s when not specified

In the validation study, 38 confirmed NEC patients (NEC I (n = 17), II (n = 11), III (n = 10)) and 13 control premature infants were matched for gestational age and sex. Baseline data did not differ significantly between the NEC and non-NEC cohorts (*P* > 0.05, Table 1).

A total of 67 neonates were enrolled in the developmental study, and 17 were excluded due to low Apgar scores or related medical history. Finally, 50 neonates were included, and 24 were eventually diagnosed with NEC. In addition, 38 confirmed NEC patients and 13 gestational age- and sex-matched non-NEC controls were enrolled in the preterm cohort.

### Increased autoantibodies in NEC

**Fig. 2:**
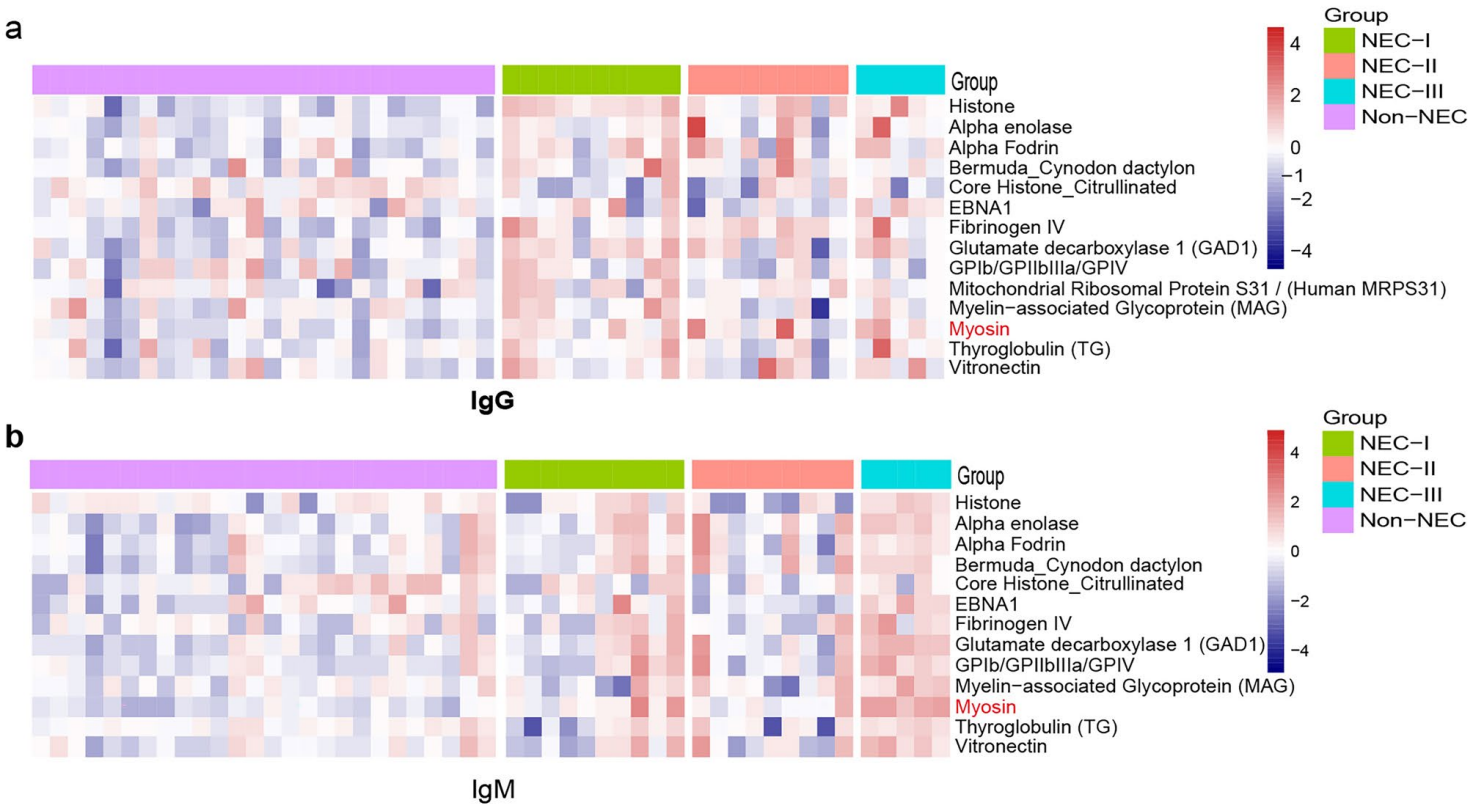
Screening of human autoantigen microarray and analysis of differential autoantibodies. **a** Array analyses confirmed higher levels of IgG autoantibodies in NEC patients. **b** Higher levels of IgM autoantibodies in NEC patients

Autoantibodies were detected using an autoantigen microarray, as reported previously [18]. First, we compared the autoantigen reactivities of patients diagnosed with NEC to those of non-NEC patients from the developmental study. The results showed that a variety of IgG and IgM autoantibodies (e.g., MAG, GAD1, myosin) in NEC patients increased compared with non-NEC controls (Fig. 2a, b). These results indicate that increased autoantibodies are associated with NEC.

A one‐way analysis of covariance (ANCOVA) was conducted to compare anti-myosin autoantibodies between cohorts, while controlling for gestational age. The results shown in Supplementary Table 1 indicate that after eliminating the influence of gestational age, there were still significant differences in anti-myosin IgG and IgM between the cohorts (*P* = 0.004, *P* < 0.001).

### Serum anti-myosin autoantibodies can serve as a diagnostic marker for NEC

**Fig. 3:**
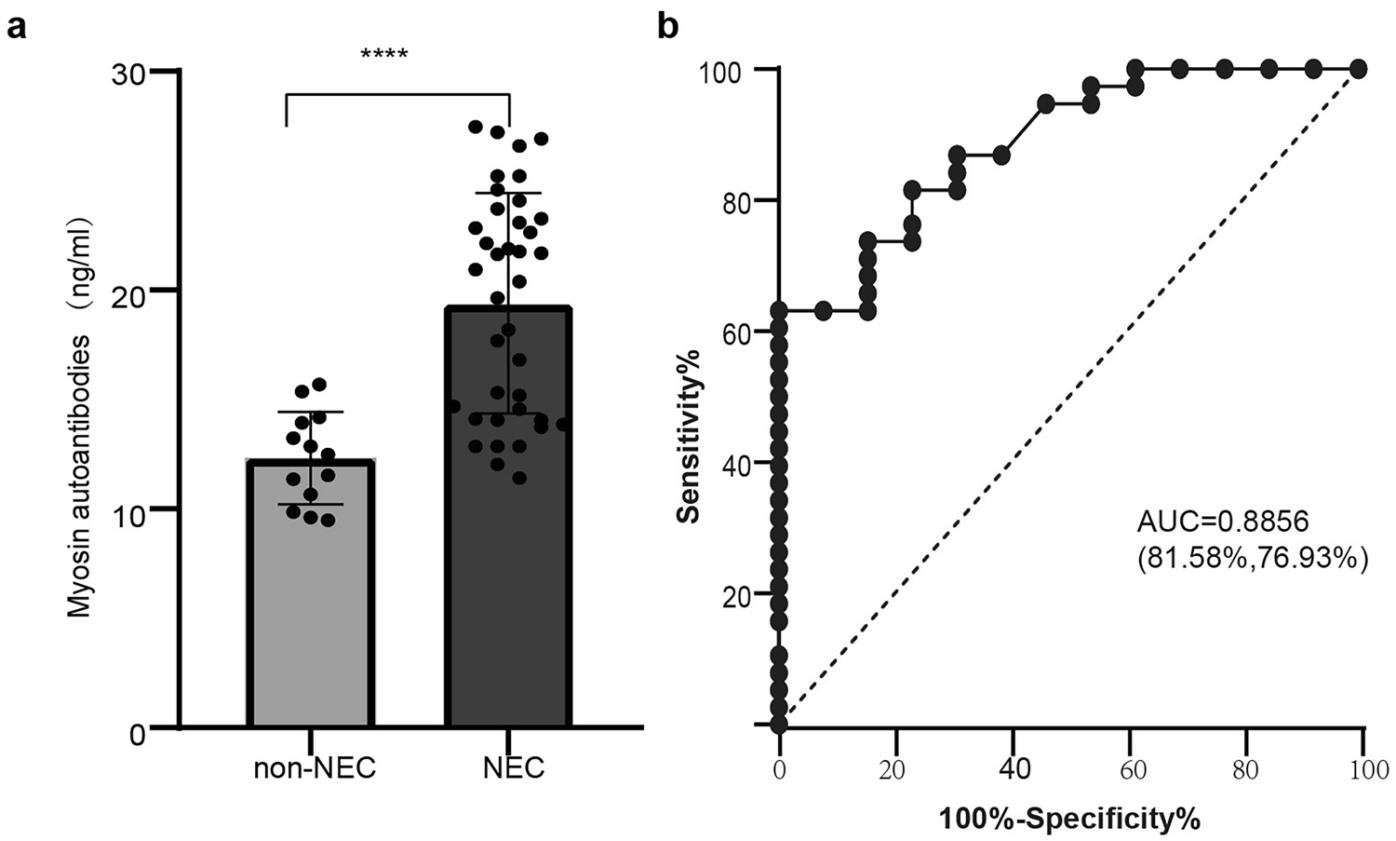
Anti-myosin autoantibodies increased in NEC plasma. **a** The level of anti-myosin autoantibodies in the NEC cohort was compared with that in the non-NEC cohort. **b** The diagnostic value of anti-myosin autoantibodies in NEC was analyzed by ROC curve analysis. **P < 0.01 indicates statistical significance, and all *P* values were tested by two-sided tests

Myosin autoantibodies may be caused by toxins, ischaemia, or inflammation [12]. We performed a validation study to verify the diagnostic value of anti-myosin autoantibodies for NEC, including 38 confirmed NEC cases and 13 gestational age- and weight-matched control premature infants without NEC. Plasma levels of anti-myosin autoantibodies were detected by enzyme-linked immunosorbent assay (ELISA). Anti-myosin autoantibodies in the plasma of NEC patients were significantly higher than those in the control subjects (*P* < 0.0001) (Fig. 3a). Anti-myosin autoantibodies were able to diagnose NEC, with an AUC of 0.8856 and an optimal cut-off value of 14.68 ng/ml (sensitivity of 81.58% and specificity of 76.93%) (Fig. 3b). These results suggest that anti-myosin autoantibodies can effectively diagnose NEC in a preterm cohort.

### Performance of serum anti-myosin autoantibodies as a biomarker for early diagnosis of NEC

**Fig. 4:**
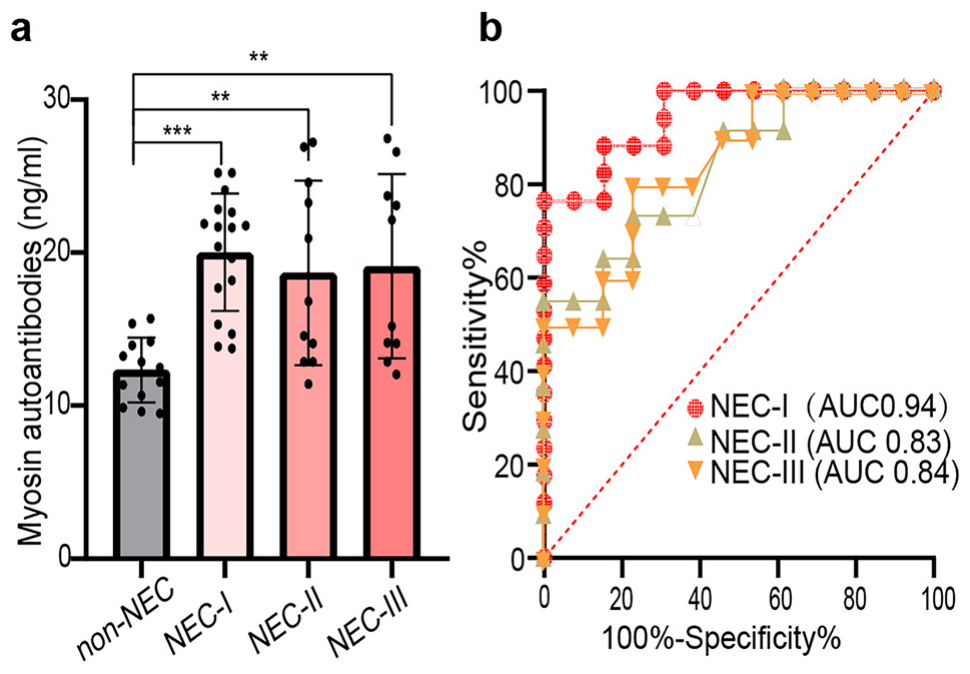
Diagnostic value of anti-myosin autoantibodies in subgroups of NEC. **a** The level of anti-myosin autoantibodies in each NEC subgroup was higher than that in the non-NEC cohort. **b** ROC curve showing the diagnostic value of anti-myosin autoantibodies for each subgroup of NEC. **P < 0.01 indicates statistical significance, and all *P* values were tested by two-sided tests

The NEC patients in the validation study were divided into three subgroups according to bell-NEC staging: stages I, II and III . Anti-myosin autoantibodies in all three NEC subgroups were significantly higher than those in the control cohort, with the most significance in NEC stage I (Fig. 4a). The diagnostic performance of anti-myosin autoantibodies was 0.9457 (95% CI: 0.8727 to 1.000) for NEC stage I, 0.8322 (95% CI: 0.6697 to 0.9947) for NEC stage II, and 0.8423 (95% CI: 0.6824 to 1.000) for NEC stage III (Fig. 4b). Therefore, anti-myosin autoantibodies have good diagnostic value for each stage of NEC, especially for stage I.

## Discussion

The pathologic characteristics of NEC embody defects in the intestinal epithelial barrier and overwhelming inflammation [5]. Early and reliable diagnosis of NEC is crucial for providing adequate treatment. However, it remains challenging due to an overlap of signs and symptoms with other gastrointestinal disorders [3]. Early symptoms of NEC, such as abdominal distension and bloody stool, are nonspecific and difficult to distinguish from other digestive diseases, and NEC progresses rapidly to intestinal necrosis and perforation.

Autoantibodies were caused by intestinal damage and exposure to self-antigens. Autoantibodies against the protein C receptor were reported for diagnosing inflammatory bowel disease [10]. Livanos et al. reported that Anti-αvβ6 autoantibodies can predict ulcerative colitis (UC) development, with an AUC of at least 0.8 up to 10 years before diagnosis [19]. In addition, a perturbed gut microbiota may also lead to an erroneous immune response due to pro-inflammatory pathways that provoke different autoimmune processes [20]. Autoantigen microarrays have been widely used for identifying autoimmune diseases, infectious diseases, and various immune disorders [18]. However, the role of autoimmune pathology in NEC development remains to be investigated. Interestingly, we confirmed that anti-myosin autoantibodies can be viewed as a reliable marker in the early stage of NEC, suggesting that autoimmune might play a role in the pathogenesis of NEC. We will collect comprehensive clinical data on autoimmune inflammatory conditions, which might provide more evidence for exploring the relationship between autoimmunity and NEC.

Myosin is a hexameric ATPase cellular motor protein abundant in smooth muscle, with these motors powering diverse cellular functions such as cytokinesis, membrane trafficking, organelle movements, and cellular migration [21]. Myosin has been demonstrated to be closely related to the integrity of the intestinal mucosal barrier [22]. In the early stage of intestinal inflammation, increased myosin light chain kinase (MLCK) induces intestinal epithelial tight junction barrier loss and increased intestinal permeability by phosphorylating myosin [22]. It has been reported that anti-myosin cross-reacts with the β-adrenergic receptor (β-AR) and triggers cAMP-dependent protein kinase A (PKA) signaling, thus leading to cell death in cardiomyopathies and myocarditis [13, 23]. Whether there is a similarity to NEC remains to be verified.

Diagnosis of NEC mainly depends on clinical manifestations and radiological examination, such as abdominal X-ray or ultrasound. However, its diagnosis is often delayed until typical pathological features occur. Several biomarkers have been proposed to improve the diagnosis of NEC, such as fatty intestinal acid-binding protein (I-FABP), trefoil factor-3 (TFF-3), and serum amyloid A (SAA) [5, 24, 25]. However, their clinical application has been hampered by the pooled sensitivity and moderate accuracy, particularly in early diagnosis [24]. It is generally agreed that abdominal distension and haematochezia are early symptoms in neonates developing NEC. Therefore, we enrolled our developmental study in neonates with suspected NEC symptoms of sudden abdominal distension and haematochezia. And both the developmental and validation studies included only blood samples taken within 48 h of the onset of NEC. Our prospective studies supported that the concentration of anti-myosin autoantibodies could benefit the early diagnosis of NEC. In the validation study, a perfect discrimination between NEC I and non-NEC was achieved, with an AUC of 0.9457. Thus, anti-myosin autoantibodies are a potential biomarker for the diagnosis of NEC, especially in the early stage.

This study has some limitations. First, this is a nice and innovative study but it is not a “diagnostic accuracy study” following STARD guidelines. As such, its findings on reliability, despite being produced with ROC analyses, are to be considered only preliminary and subjected to future diagnostic accuracy studies. Second, this is a single-center study, and the samples were from patients of a single ethnicity. Considering the study design of the early stage of NEC, we, respectively, enrolled only 24 and 38 patients, and the sample size of the control cohort needs to be expanded. Moreover, dynamic analysis of the variation in the acute and recovery stages is needed. Third, the role of anti-myosin autoantibodies in developing NEC remains unclear. Digging deeper into the mechanism of anti-myosin autoantibodies can help us to better understand the pathogenesis of NEC.

In conclusion, our study suggests that anti-myosin autoantibodies can serve as an efficient biomarker for NEC diagnosis. Moreover, there was a significant increase in anti-myosin autoantibodies during the first stage of NEC. Finally, considering the relationship of anti-myosin autoantibodies with the immune system may help to clarify the pathogenesis of NEC, opening up new therapeutic perspectives for this severe neonatal disease.

### Supplementary Information

Below is the link to the electronic supplementary material.

Supplementary file1 (DOCX 479 KB)

Supplementary file2 (XLSX 9 KB)

## Data Availability

All data included in this study are available upon request by contacting with the corresponding author.

## Abbreviations

AUC: Area under the curve
CI: Confidence interval
ELISA: Enzyme-linked immunosorbent assay
I-FABP: Fatty intestinal acid-binding protein
MHC: Myosin heavy chain
MLC: Myosin light chain
MLCK: Myosin light chain kinase
NEC: Necrotizing enterocolitis
ROC: Receiver operating characteristic
PKA: Protein kinase A
SAA: Serum amyloid A
TFF-3: Trefoil factor-3
β-AR: β-Adrenergic receptor
