# Supplementary material for: Plasma anti-myosin autoantibodies in the diagnosis of necrotizing enterocolitis
Source: Eur J Pediatr. 2023 Sep 16;182(11):5203–10. doi: 10.1007/s00431-023-05188-6 (PMC10640473; doi:10.1007/s00431-023-05188-6)
Supplement: Supplementary file 1 — Supplementary file1 (DOCX 479 KB) [file 431_2023_5188_MOESM1_ESM.docx]

Plasma Anti-myosin Autoantibodies in the Diagnosis of Necrotizing Enterocolitis

Yuqiong Chen ^1,3†^, Chaoting Lan ^2†^, Weiyong Zhong ^2†^, Kai Song^2^, Zuyi Ma^2^, Lihua Huang^2*^, Yun Zhu^2*^, Huimin Xia ^1,2*^

† These authors have contributed equally to this work and share first authorship.

^1^ Department of Pediatrics, The First Affiliated Hospital of Jinan University, Guangzhou, Guangdong, China

^2^ Provincial Key Laboratory of Research in Structure Birth Defect Disease and Department of Pediatric Surgery, Guangzhou Women and Children’s Medical Center, Guangzhou Medical University, Guangzhou, Guangdong, China

^3^ Department of Pediatrics, The First Hospital of Chenzhou, Chenzhou, Hunan, China

*** Correspondence:**Lihua Huang

[hlh2208@126.com](mailto:hlh2208@126.com)， Guangzhou Women and Children’s Medical Center, No.9 Jinsui Road, Zhujiang New Town, Tianhe District, Guangzhou, Guangdong, CN 510623

Yun Zhu
zhuyun210@163.com, Guangzhou Women and Children’s Medical Center, No.9 Jinsui Road, Zhujiang New Town, Tianhe District, Guangzhou, Guangdong, CN 510623

Huimin Xia
[xia-huimin@foxmail.com](mailto:xia-huimin@foxmail.com), The First Affiliated Hospital of Jinan University, No. 613 West Huangpu Avenue, Tianhe District, Guangzhou, Guangdong, CN 510630

Abstract

Purpose: We aimed to assess whether autoantibodies can be used as biomarkers for necrotizing enterocolitis (NEC) and applied for its early diagnosis.

Methods: A prospective observational study was conducted in neonates with suspected NEC abdominal distension (the developmental study), which consisted of 50 neonates finally divided into NEC (n=24) and non-NEC (n=26) cohorts based on follow-up results. Serum samples were collected within 48 hours of illness onset and used for screening NEC-associated plasma autoantibodies by autoantigen microarray. Additionally, we validated anti-myosin autoantibodies by enzyme-linked immunosorbent assay (ELISA) in an independent validation sdudy, for which we selected plasma samples within 48 hours of onset of NEC (n=38) and samples of gestational age- and weight-matched controls (n=13).

Results: Autoantigen microarray revealed that both IgG and IgM anti-myosin autoantibodies in plasma from neonates with NEC were significantly higher than those in neonates with other diagnoses. ELISA showed that plasma anti-myosin autoantibodies increased in the NEC **cohort**, with 1.5-fold higher levels than in the non-NEC **cohort**. Anti-myosin autoantibodies were able to distinguish NEC from non-NEC, achieving an area under the curve (AUC) of 0.8856 (95% confidence interval [CI]: 0.7918-0.9795), with sensitivity of 81.58% and specificity of 76.93%. Plasma anti-myosin autoantibodies were significantly higher in all three subtypes of NEC (*P* <0.0001 for NEC Ⅰ; *P* =0.0018 for NEC Ⅱ; *P* =0.0011 for NEC Ⅲ), especially in NEC stage I, than that in the non-NEC controls.

Conclusion: Anti-myosin autoantibodies may be applied as a promising diagnostic marker for NEC, especially for NEC stage I.

Keywords: Necrotizing enterocolitis; diagnosis; autoantibody; myosin

**What is Known：**

- *Intestinal damage and self-antigen exposure may lead to increased autoantibodies, and they are widely used as biomarkers for diagnosing inflammatory bowel disease.*
- *Necrotizing enterocolitis (NEC) is a devastating disease with overwhelming inflammation and immune dysregulation.*

**What is New：**

- *Increased autoantibodies were present in patients with NEC, even before typical X-ray manifestations.*
- *Anti-myosin autoantibodies may be applied as a promising diagnostic marker for NEC.*

**List of abbreviations**

| Abbreviation | Full name |
| --- | --- |
| AUC | area under the curve |
| CI | confidence interval |
| ELISA | Enzyme-linked immunosorbent assay |
| I-FABP | fatty intestinal acid-binding protein |
| MHC | myosin heavy chain |
| MLC | myosin light chain |
| MLCK | myosin light chain kinase |
| NEC | Necrotizing enterocolitis |
| ROC | receiver operating characteristic |
| PKA | protein kinase A |
| SAA | serum amyloid A |
| TFF-3 | trefoil factor-3 |
| β-AR | β-adrenergic receptor |

**Introduction**

Necrotizing enterocolitis (NEC) is a devastating disease with high morbidity and mortality (15% to 30%) among preterm infants [1]. Early symptoms of NEC, such as abdominal distension and [haematochezia](javascript:;), are nonspecific but progress rapidly, with intestinal necrosis and perforation [2]. Early diagnosis is challenging due to the overlap of signs and symptoms with other gastrointestinal disorders [1; 3]. The presence of portal venous gas in abdominal X-ray is a typical imaging feature of NEC, but it occurs in Bell stage Ⅱ or more [4]. Because of the limited diagnostic accuracy of laboratory tests and delayed imaging modalities, biomarkers that can sensitively identify infants at risk of developing NEC are needed.

The pathologic characteristics of NEC include defects in the intestinal epithelial barrier and overwhelming inflammation [5; 6]. Intestinal immaturity, mucosal damage, pathogenic microbial invasion, and inappropriate feeding can all lead to intestinal damage and an inflammatory cascade in newborns [7]. Hypoxia and infection in utero or during birth also increase the risk of NEC [8]. Previous studies have shown that damage to tissue cells can cause the production of autoantibodies [9]. Autoimmunity has been verified in inflammatory bowel disease and immune-mediated inflammatory diseases, and has been linked to disease severity and activity [10]. However, the role of autoimmunity in the development of NEC remains unknown.

Myosin, a hexameric ATPase cellular motor protein with two heavy chains (MHCs) and four light chains (MLCs), is a sarcolemmal protein involved in the process of muscular contraction [11]. Myosin exists inside cells, and tissue damage and exposure to self-antigens may be caused by toxins, ischaemia, or inflammation [12]. Anti-myosin autoantibodies are widely used as diagnostic biomarkers, especially in heart diseases such as myocarditis [13], dilated cardiomyopathy [14], Kawasaki disease [15], rheumatic fever [16] and myocardial ischaemia [17]. Intestinal injury, inflammation, and necrosis are important features of NEC. Therefore, we hypothesize that autoantibodies produced by antigen exposure are involved in **the** development of NEC.

In this study, we screened for activated autoantibodies using an autoantigen microarray in neonates with suspected NEC abdominal distension (the developmental study). We found that both IgG and IgM among anti-myosin autoantibodies in plasma from neonates with NEC were significantly higher than in neonates with other diagnoses. We speculated that elevated anti-myosin autoantibodies may predict **the** risk of developing NEC at an early stage. To assess whether anti-myosin autoantibodies can diagnose NEC in preterm infants, we measured concentrations of serum anti-myosin autoantibodies in an independent validation sdudy. The results showed that anti-myosin autoantibodies are able to distinguish NEC from non-NEC, achieving an area under the curve (AUC) of 0.8856, with an AUC of 0.9457 for NEC stage I. Our data suggest that anti-myosin autoantibodies may serve as a biomarker for the diagnosis of NEC, especially in NEC stage I.

**Materials and methods**

**Subjects**

Both the developmental study (NEC=24; non-NEC=26) and the validation sdudy (NEC=38; non-NEC=13) were enroled in 2019 and 2020, and NEC cohorts with onset time less than 48 hours were enroled . Both sdudies are prospective. According to Bell staging criteria [1], **the** diagnosis of NEC was confirmed by more than three neonatologists and neonatal surgeons, who were blinded to autoantibodies testing. The study was approved by the Medical Ethics Committee of Guangzhou Women and Children’s Medical Center (No. 2018052406), and written consent was obtained from the participant's parents or legal guardian.

The developmental study included consecutively admitted neonates with sudden abdominal distension and a possible clinical result of NEC, and the serum samples was used to test for IgM and IgG autoantibodies. Eligible newborns were identified by neonatologists who were unaware of the study aims, with the following criteria fulfilled: (1) premature infants with a corrected gestational age of less than 44 weeks; (2) sudden abdominal distension, with haematochezia or positive faecal occult blood and, onset time less than 48 hours; and (3) parental consent obtained. Newborns with the following conditions were excluded: (1) Apgar score <5 at 5 minutes; (2) congenital malformations or inborn errors of metabolism; (3) maternal history of autoimmune diseases; and (4) incomplete clinical data. Finally, 50 neonates were included, and 24 were eventually diagnosed with NEC.

Additionally, the validation sdudy was independent; samples were obtained from the hospital's biobank, from which we selected plasma samples of diagnosed NEC preterm patients. And we only included plasma samples within 48 hours of the onset of NEC (n=38). NEC patients were stratified according to gestational age at birth, and then birth weight, age at time of blood test, and sex-matched preterm controls were selected, finally including 13 matched controls. The exclusion criteria were the same as those for the developmental study.

**Data collection and clinical assessment**

Patient demographic and clinical information, including gestational age, birth weight, sex, delivery mode, fetal distress, feeding, diagnosis and treatment, were obtained from electronic medical records. Clinical information was collected and checked by 2 independent clinicians. To avoid information bias, the clinicians **were** blinded to the subsequent autoantibody tests.

Additionally, blood specimens for autoantigen microarray were collected in EDTA vacuum tubes, and plasma was obtained by centrifugation at 1,500 × rpm for 20 min at 4 °C within 2 h and again at 3,000 × rpm for 15 min. The collected plasma was stored at -80 °C. Blood samples for ELISA were peripheral blood samples taken for routine blood tests when development of NEC was first suspected.

**Autoantigen microarray**

We used an autoantigen microarray **composed** of 48 autoantigens and 8 calibration proteins to screen for human autoimmune antigen microarrays. The autoantigen microarrays were manufactured, hybridized, and scanned as previously described [18]. IgG and IgM autoantibody tests were performed at Yijin Biotechnology Co., Ltd.

**ELISA**

Quantitative sandwich enzyme immunoassays for human anti-myosin heavy chain autoantibodies (MYHA) were performed as recommended by the manufacturer (Shanghai Zhenke Biotechnology Co., Ltd., ZK-15613).

**Statistical analysis**

Statistical analysis of the data was conducted by employing R and GraphPad Prism 8.0 (GraphPad Software Inc., CA, USA). A normal distribution of the variables was tested using the Shapiro–Wilk test. Normalized data are described by the mean and standard deviation. Quantitative variables were analyzed utilizing the *t* test, and grade data were assessed using chi-square tests. Analysis of covariance models **was** used when comparing continuous variables adjusting for potential confounders. The significance level was set at *P* < 0.05, and all *P* values were obtained from two-sided tests.

For autoantigen microarray analysis, normalization was executed by implementing the robust-linear-mode (RLM) method. M-statistics were applied for differential markers detection. The differential markers were subjected to cluster analysis by R package of “pheatmap”.

A receiver operating characteristic (ROC) curve provided the AUC for diagnostic value analysis. Diagnostic accuracy was evaluated through sensitivity and specificity measurements.

**Results**

**Characteristics of the subjects**

In the developmental study, a total of 67 neonates were enroled, and 17 were excluded due to low Apgar scores or related medical history. Finally, 50 neonates were included, and 24 were eventually diagnosed with NEC. They were further classified into Bell's stage Ⅰ (n=10), Ⅱ (n=9), and Ⅲ (n=5). The remaining 26 patients were assigned to the non-NEC **cohort**, and they were diagnosed with [feeding intolerance](javascript:;) (n=13, including milk protein allergies and [lactose intolerance](javascript:;)), Hirschsprung’s disease (n=5), intestinal malrotation (n=5), or gastrointestinal problems of unknown origin (n=3). The flow chart of subject enrolment is shown in Fig 1. There was no significant difference in birth weight, or sex, between the NEC and non-NEC **cohorts** (*P* > 0.05, Table 1), but gestational age was younger in the NEC **cohort** (30.02±1.96 vs. 31.51±2.02, *P*=0.015).

**
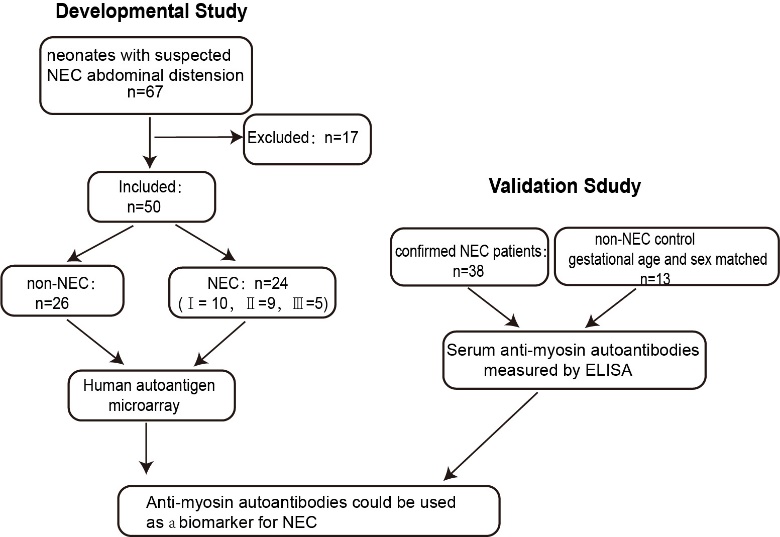
**In the validation sdudy, 38 confirmed NEC patients (NEC Ⅰ (n=17), Ⅱ (n=11), Ⅲ (n=10)) and 13 control premature infants were matched for gestational age and sex. Baseline data did not differ significantly between the NEC and non-NEC **cohorts** (*P* > 0.05, Table 1).

**Fig. 1 Flow chart of subject enrolment**

A total of 67 neonates were enroled in the developmental study, and 17 were excluded due to low Apgar scores or related medical history. Finally, 50 neonates were included, and 24 were eventually diagnosed with NEC. In addition, 38 confirmed NEC patients and 13 gestational age- and sex-matched non-NEC controls were enroled in the preterm cohort.

| **Table 1 Patient Characteristics of Clinical Cases** | | | | | | |
| --- | --- | --- | --- | --- | --- | --- |
|  | Developmental Study | | | Validation Sdudy | | |
|  | non-NEC | NEC | *P* | non-NEC | NEC | *P* |
| Gestational age(weeks) | 31.51±2.02 | 30.02±1.96 | 0.015 | 30.21±1.93 | 29.61±1.525 | 0.323 |
|  |  |  |  |  |  |  |
| Birth weight(g) | 1403.85±288.85 | 1279.17±280.51 | 0.129 | 1159.23±174.62 | 1177.63±223.32 | 0.763 |
|  |  |  |  |  |  |  |
| Sex[male，N (%)] | 18(69.2) | 14(58.3) | 0.423 | 7（53.8） | 25（65.8） | 0.442 |
| Cesarean section[N (%)] | 16(61.5) | 15(62.5) | 0.944 | 8（61.5） | 21（55.3） | 0.693 |
| Apgar score at 5min | 9.23±0.951 | 9±0.78 | 0.355 | 9.54±0.52 | 9.45±0.83 | 0.712 |
|  |  |  |  |  |  |  |
| Antenatal steroids^a^ [N (%)] | 13（50.0） | 16（66.7） | 0.233 | 9（69.2） | 30（78.9） | 0.476 |
|  |  |  |  |  |  |  |
| PROM^b^>18h[N (%)] | 7(26.9) | 7(29.2) | 0.86 | 3（23.1） | 9（23.7） | 0.964 |
| Intrapartum antibiotic prophylaxis for PROM [N (%)] | 5（19.2） | 4（16.7） | 0.814 | 2（15.4） | 7（18.4） | 0.804 |
|  |  |  |  |  |  |  |
| Breast Feeding[N (%)] | 20(76.9) | 19(79.2) | 0.848 | 10（76.9） | 30（78.9） | 0.878 |
| Early-onset sepsis[N (%)] | 1（3.8） | 2（8.3） | 0.504 | 0（0） | 1（2.6） | 0.555 |
|  |  |  |  |  |  |  |
| Late-onset sepsis[N (%)] | 0(0) | 1 | 0.293 | 1（7.7） | 2（5.3） | 0.748 |
| Age（day） | 22.15±12.18 | 28.67±20.79 | 0.189 | 23.85±13.54 | 24.03±13.52 | 0.967 |
| Total | 26 | 24 |  | 13 | 38 |  |
| ^a^Intramuscular steroid cycle in two doses of 12mg over a 24-h period;^b^PROM, prelabor rupture of membranes; ^c^Breast milk feeding means breast milk>50%.NEC, Necrotizing enterocolitis. Data were expressed as x̅±s when not specified. | | | | | | |
|  |  |  |  |  |  |  |

**Increased autoantibodies in NEC**

Autoantibodies were detected using an autoantigen microarray, as reported previously [18]. First, we compared autoantigen the reactivities of patients diagnosed with NEC to non-NEC from the developmental study. The results showed that a variety of IgG and IgM autoantibodies (e.g., MAG, GAD1, myosin) in NEC patients increased compared with non-NEC controls (Fig.2a and b). These results indicate that increased autoantibodies are associated with NEC.


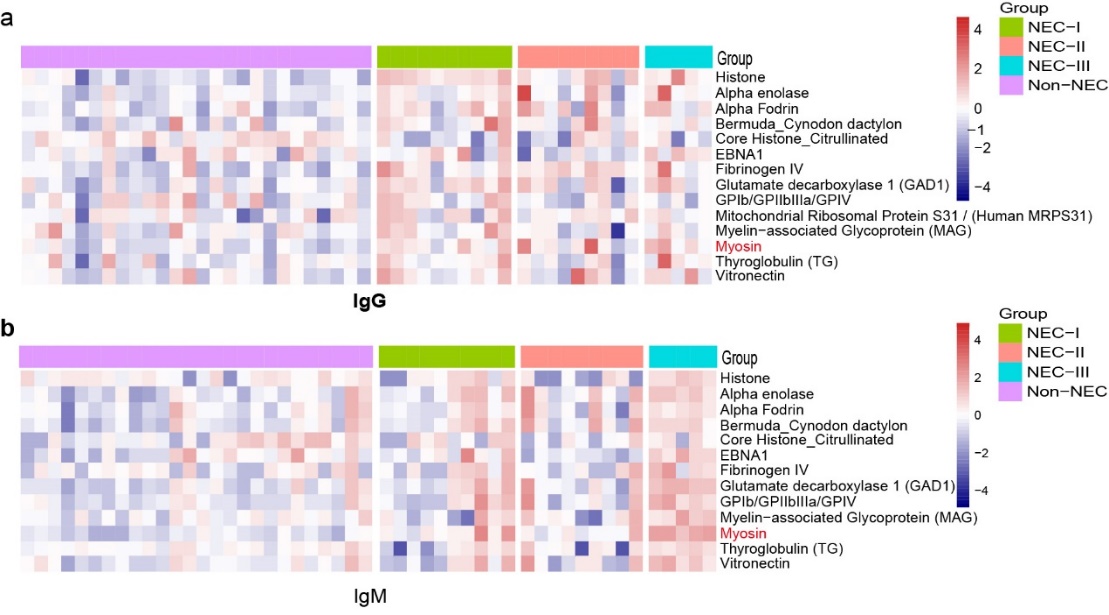


**Fig. 2 Screening of human autoantigen microarray and analysis of differential autoantibodies**

(a) Array analyses confirmed higher levels of IgG autoantibodies in NEC patients. (b) Higher levels of IgM autoantibodies in NEC patients.

A one‐way analysis of covariance (ANCOVA) was conducted to compare anti-myosin autoantibodies between **cohorts**, while controlling for gestational age. The results shown in Supplementary Table 1 indicate that after eliminating the influence of gestational age, there were still significant differences in anti-myosin IgG and IgM between the **cohorts** (*P*=0.004, *P*<0.001).

**Serum anti-myosin autoantibodies can serve as a diagnostic marker for NEC**

Myosin autoantibodies may be caused by toxins, ischaemia, or inflammation [12]. We performed a validation study to verify the diagnostic value of anti-myosin autoantibodies for NEC, including 38 confirmed NEC cases and 13 gestational age- and weight-matched control premature infants without NEC. Plasma levels of anti-myosin autoantibodies were detected by enzyme-linked immunosorbent assay (ELISA). Anti-myosin autoantibodies in the plasma of NEC patients were significantly higher than those in the control subjects (*P* < 0.0001) (Fig. 3a). Anti-myosin autoantibodies were able to diagnose NEC, with an AUC of 0.8856 and an optimal cut-off value of 14.68 ng/ml (sensitivity of 81.58% and specificity of 76.93% )(Fig. 3b). These results suggest that anti-myosin autoantibodies can effectively diagnose NEC in a preterm cohort.


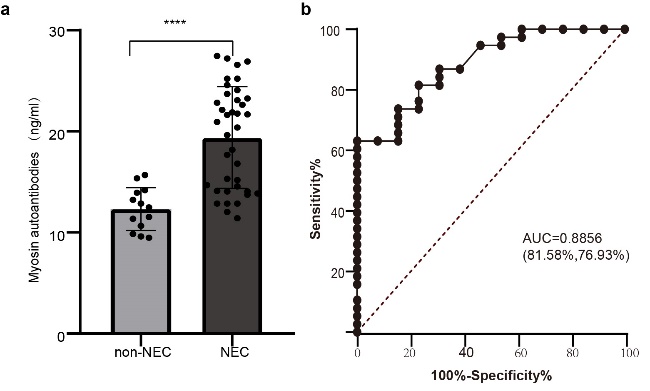


**Fig. 3 Anti-myosin autoantibodies increased in NEC plasma**

(a) The level of anti-myosin autoantibodies in the NEC **cohort** was compared with that in the non-NEC **cohort**. (b) The diagnostic value of anti-myosin autoantibodies in NEC was analysed by ROC curve analysis. “** *P* < 0.01” indicates statistical significance, and all *P* values were tested by two-sided tests.

**Performance of serum anti-myosin autoantibodies as a biomarker for early diagnosis of NEC**

The NEC patients in the validation sdudy were divided into three subgroups according to Bell-NEC staging: stages Ⅰ, Ⅱ, and Ⅲ. Anti-myosin autoantibodies in all three NEC subgroups were significantly higher than those in the control cohort, with the most significance in NEC stage I (Fig. 4a). The diagnostic performance of anti-myosin autoantibodies was 0.9457 (95% CI: 0.8727 to 1.000) for NEC stage I, 0.8322 (95% CI: 0.6697 to 0.9947) for NEC stage Ⅱ, and 0.8423 (95% CI: 0.6824 to 1.000) for NEC stage Ⅲ (Fig. 4b). Therefore, anti-myosin autoantibodies have good diagnostic value for each stage of NEC, especially for stage Ⅰ.

**
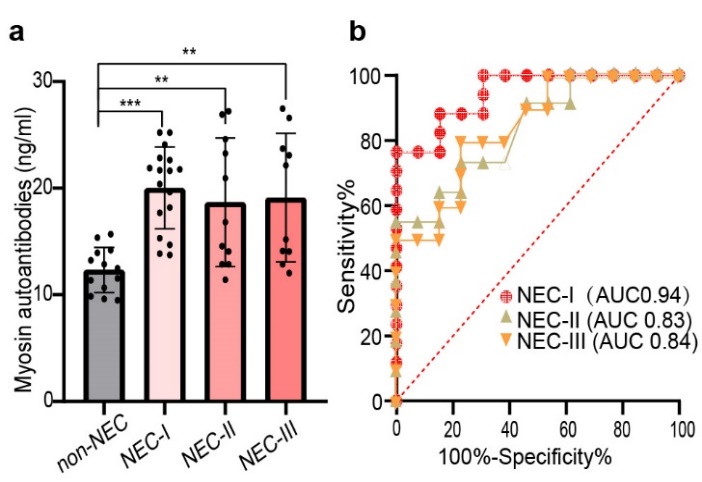
**

**Fig. 4 Diagnostic value of anti-myosin autoantibodies in subgroups of NEC**

(a) The level of anti-myosin autoantibodies in each NEC subgroup was higher than that in non-NEC cohort. (b) ROC curve showing the diagnostic value of anti-myosin autoantibodies for each subgroup of NEC. “** *P* < 0.01” indicates statistical significance, and all *P* values were tested by two-sided tests.

**Discussion**

The pathologic characteristics of NEC embody defects in the intestinal epithelial barrier and overwhelming inflammation [5]. Early and reliable diagnosis of NEC is crucial for providing adequate treatment. However, it remains challenging due to an overlap of signs and symptoms with other gastrointestinal disorders [3]. Early symptoms of NEC, such as abdominal distension and bloody stool, are nonspecific and difficult to distinguish from other digestive diseases, and NEC progresses rapidly to intestinal necrosis and perforation.

Autoantibodies were caused by intestinal damage and exposure to self-antigens. Autoantibodies against the protein C receptor were reported for diagnosing inflammatory bowel disease [10]. Livanos et al. reported **that** Anti-αvβ6 autoantibodies can predict ulcerative colitis (UC) development, with an AUC of at least 0.8 up to 10 years before diagnosis [19]. In addition, a perturbed gut microbiota may also lead to an erroneous immune response due to pro-inflammatory pathways that provoke different autoimmune processes [20]. Autoantigen microarrays have been widely used for identifying autoimmune diseases, infectious diseases and various immune disorders [18]. However, the role of autoimmune pathology in NEC development remains to be investigated. Interestingly, we confirmed that anti-myosin autoantibodies can be viewed as a reliable marker in the early stage of NEC, suggesting that autoimmune might play a role in the pathogenesis of NEC. We will collect comprehensive clinical data on autoimmune inflammatory conditions, which might provide more evidence for exploring the relationship between autoimmunity and NEC.

Myosin is a hexameric ATPase cellular motor protein abundant in smooth muscle, with these motors powering diverse cellular functions such as cytokinesis, membrane trafficking, organelle movements, and cellular migration [21]. Myosin has been demonstrated to be closely related to the integrity of the intestinal mucosal barrier [22]. In the early stage of intestinal inflammation, increased myosin light chain kinase (MLCK) induces intestinal epithelial tight junction barrier loss and increased intestinal permeability by phosphorylating myosin [22]. It has been reported that anti-myosin cross-reacts with the β-adrenergic receptor (β-AR) and triggers cAMP-dependent protein kinase A (PKA) signaling, thus leading to cell death in cardiomyopathies and myocarditis [13; 23]. Whether there is a similarity to NEC remains to be verified.

Diagnosis of NEC mainly depends on clinical manifestations and [radiological examination](javascript:;), such as abdominal X-ray or ultrasound. However, its diagnosis is often delayed until typical pathological features occur. Several biomarkers have been proposed to improve **the** diagnosis of NEC, such as fatty intestinal acid-binding protein (I-FABP), trefoil factor-3 (TFF-3), and serum amyloid A (SAA) [5; 24; 25]. However, their clinical application has been hampered by the pooled sensitivity and moderate accuracy, particularly in early diagnosis [24]. It is generally agreed that abdominal distension and haematochezia are early symptoms in neonates developing NEC. Therefore, we enroled our developmental study in neonates with suspected NEC symptoms of sudden abdominal distension and haematochezia. And both the developmental and validation studies included only blood samples taken within 48 hours of **the** onset of NEC. Our prospective studies supported that the concentration of anti-myosin autoantibodies could benefit the early **diagnosis** of NEC. In **the** validation study, a perfect discrimination between NEC Ⅰ and non-NEC was achieved, with an AUC of 0.9457. Thus, anti-myosin autoantibodies are a potential biomarker for the diagnosis of NEC, especially in the early stage.

This study has some limitations. First,  this is a nice and innovative study but it is not a "diagnostic accuracy study" following STARD guidelines. As such its findings on reliability, despite being produced with ROC analyses are to be considered only preliminary and subjected to future diagnostic accuracy studies. Second, this is a single-center study, and the samples were from patients of a single ethnicity. Considering the study design of the early stage of NEC, we [respectively](javascript:;) enroled only 24 and 38 patients, and the sample size of the control **cohort** needs to be expanded. Moreover, dynamic analysis of the variation in the acute and recovery stages is needed. Third, the role of anti-myosin autoantibodies in developing NEC remains unclear. Digging deeper into the mechanism of anti-myosin autoantibodies can help us to better understand the pathogenesis of NEC.

In conclusion, our study suggests that anti-myosin autoantibodies can serve as an efficient biomarker for NEC diagnosis. Moreover, there was a significant increase in anti-myosin autoantibodies during the first stage of NEC. Finally, considering the relationship of anti-myosin autoantibodies with the immune system may help to clarify the pathogenesis of NEC, opening up new therapeutic perspectives for this severe neonatal disease.

**References**

1. Lin PW, Stoll BJ (2006) Necrotising enterocolitis. Lancet 368:1271-1283

2. Eaton S (2017) Necrotizing enterocolitis symposium: Epidemiology and early diagnosis. J Pediatr Surg 52:223-225

3. Kim JH, Sampath V, Canvasser J (2020) Challenges in diagnosing necrotizing enterocolitis. Pediatr Res 88:16-20

4. Esposito F, Mamone R, Di Serafino M, Mercogliano C, Vitale V, Vallone G, Oresta P (2017) Diagnostic imaging features of necrotizing enterocolitis: a narrative review. Quant Imaging Med Surg 7:336-344

5. Carroll D, Corfield A, Spicer R, Cairns P (2003) Faecal calprotectin concentrations and diagnosis of necrotising enterocolitis. Lancet 361:310-311

6. Mihi B, Lanik WE, Gong Q, Good M (2021) A Mouse Model of Necrotizing Enterocolitis. Methods Mol Biol 2321:101-110

7. Hackam DJ, Sodhi CP (2022) Bench to bedside - new insights into the pathogenesis of necrotizing enterocolitis. Nat Rev Gastroenterol Hepatol 19:468-479

8. Blakely ML, Tyson JE, Lally KP, Hintz SR, Eggleston B, Stevenson DK, Besner GE, et al. (2021) Initial Laparotomy Versus Peritoneal Drainage in Extremely Low Birthweight Infants With Surgical Necrotizing Enterocolitis or Isolated Intestinal Perforation: A Multicenter Randomized Clinical Trial. Ann Surg 274:e370-e380

9. Xiao ZX, Miller JS, Zheng SG (2021) An updated advance of autoantibodies in autoimmune diseases. Autoimmun Rev 20:102743

10. Kakuta Y, Shirai T, McGovern DPB, Braun J, Fujii H, Masamune A (2023) Novel Diagnostic Autoantibodies Against Endothelial Protein C Receptor in Patients With Ulcerative Colitis. Clin Gastroenterol Hepatol 21:844-846

11. Pette D, Staron RS (2000) Myosin isoforms, muscle fiber types, and transitions. Microsc Res Tech 50:500-509

12. Nussinovitch U, Shoenfeld Y (2013) The clinical and diagnostic significance of anti-myosin autoantibodies in cardiac disease. Clin Rev Allergy Immunol 44:98-108

13. Buggey J, ElAmm CA (2018) Myocarditis and cardiomyopathy. Curr Opin Cardiol 33:341-346

14. Dandel M, Wallukat G, Potapov E, Hetzer R (2012) Role of beta(1)-adrenoceptor autoantibodies in the pathogenesis of dilated cardiomyopathy. Immunobiology 217:511-520

15. Kobayashi H, Kimura MY, Hasegawa I, Suganuma E, Ikehara Y, Azuma K, Ito T, Ebata R, Kurashima Y, Kawasaki Y, Shiko Y, Saito N, Iwase H, Lee Y, Noval Rivas M, Arditi M, Zuka M, Hamada H, Nakayama T (2022) Increased Myosin light chain 9 expression during Kawasaki disease vasculitis. Front Immunol 13:1036672

16. Garcia AF, Yamaga KM, Shafer LA, Bollt O, Tam EK, Cunningham MW, Kurahara DK (2016) Cardiac Myosin Epitopes Recognized by Autoantibody in Acute and Convalescent Rheumatic Fever. Pediatr Infect Dis J 35:1021-1026

17. Galvin JE, Hemric ME, Kosanke SD, Factor SM, Quinn A, Cunningham MW (2002) Induction of myocarditis and valvulitis in lewis rats by different epitopes of cardiac myosin and its implications in rheumatic carditis. Am J Pathol 160:297-306

18. Yeste A, Quintana FJ (2013) Antigen microarrays for the study of autoimmune diseases. Clin Chem 59:1036-1044

19. Livanos AE, Dunn A, Fischer J, Ungaro RC, Turpin W, Lee SH, Rui S, et al. (2023) Anti-Integrin alphavbeta6 Autoantibodies Are a Novel Biomarker That Antedate Ulcerative Colitis. Gastroenterology 164:619-629

20. Shaheen WA, Quraishi MN, Iqbal TH (2022) Gut microbiome and autoimmune disorders. Clin Exp Immunol 209:161-174

21. Sweeney HL, Houdusse A, Robert-Paganin J (2020) Myosin Structures. Advances in experimental medicine and biology 1239:7-19

22. Odenwald MA, Turner JR (2013) Intestinal permeability defects: is it time to treat? Clin Gastroenterol Hepatol 11:1075-1083

23. Ko YT, Hartner WC, Kale A, Torchilin VP (2009) Gene delivery into ischemic myocardium by double-targeted lipoplexes with anti-myosin antibody and TAT peptide. Gene Ther 16:52-59

24. Cheng S, Yu J, Zhou M, Tu Y, Lu Q (2015) Serologic Intestinal-Fatty Acid Binding Protein in Necrotizing Enterocolitis Diagnosis: A Meta-Analysis. Biomed Res Int 2015:156704

25. Ng PC (2018) An update on biomarkers of necrotizing enterocolitis. Semin Fetal Neonatal Med 23:380-386

**Statements and Declarations**

**Funding**

Huimin Xia thanks the support of the Science and Technology Planning Project of Guangdong Province (Grant No. 2019B020227001) and the Science and Technology Project of Guangzhou (Grant No.202206080002). Lihua Huang thanks the grant National Natural Science Foundation of China (Grant NO. 82070528). Yuqiong Chen thanks the Science and Technology Project of Hunan (Grant No. 2021JJ40005) and Science Research Program of Hunan Provincial Health Commission (202106030771). Chaoting Lan is supported by the China Postdoctor Science Foundation (Grant NO. 2023M730791) and the Research Foundation of Guangzhou Women and Children’s Medical Center for Clinical Doctor (Grant NO. 2023BS015).

**Competing Interests**

The authors have no conflicts of interest to declare.

**Author Contributions**

Huimin Xia and Yun Zhu contributed to the conception and design of the study. Yun Zhu organized the database. Yuqiong Chen and Chaoting Lan wrote the original draft of the manuscript. Lihua Huang edited the manuscript. Weiyong Zhong, Chaoting Lan, Yuqiong Chen, Kai Song, Zuyi Ma contributed to the sample and data collection. Weiyong Zhong and Kai Song performed the statistical analysis. All authors approved the submitted version.

**Ethics approval**

This study was performed in line with the principles of the Declaration of Helsinki. Approval was granted by the Ethics Committee of Guangzhou Women and Children’s Medical Center (No. 2018052406)*.*

**Consent to participate**

Written consent was obtained from the participant's parents or legal guardian.

**Consent to publish**

Not applicable.

**Availability of data and materials**

All data included in this study are available upon request by contacting with the corresponding author.

**Acknowledgements**

We are grateful to the participants and their guardians for their support of this study.
